# First Report of *Kosakonia radicincitans* Bacteraemia from Europe (Austria) - Identification and Whole-Genome Sequencing of Strain DSM 107547

**DOI:** 10.1038/s41598-020-58689-x

**Published:** 2020-02-06

**Authors:** Tanja Mertschnigg, Sascha Patz, Matthias Becker, Gebhard Feierl, Silke Ruppel, Boyke Bunk, Cathrin Spröer, Jörg Overmann, Gernot Zarfel

**Affiliations:** 1 Institute of Hygiene, Microbiology and Environmental Medicine, Medical University of Graz, Graz, Austria; 2 Algorithms in Bioinformatics, Center for Bioinformatics, University of Tübingen, Tübingen, Germany; 3 Institute for National and International Plant Health, Julius Kühn Institute, Federal Research Centre for Cultivated Plants, Braunschweig, Germany; 4 Leibniz Institute of Vegetable and Ornamental Crops, Grossbeeren, Germany; 5 Leibniz Institute DSMZ-German Collection of Microorganisms and Cell Cultures, Braunschweig, Germany; 6 Microbiology, Braunschweig University of Technology, Braunschweig, Germany

**Keywords:** Bacteriology, Bacterial infection

## Abstract

*Kosakonia radicincitans* is a species within the new genus *Kosakonia*. Many strains of this genus have been isolated from plants, but some strains are assumed to act as facultative human pathogens. In this study, an in-depth analysis of a *Kosakonia* isolate from human blood was performed. The strain was originally isolated from blood and identified as a member of the *Enterobacter cloacae* complex, exhibiting an atypical result in susceptibility testing. Therefore, the genetic background was examined, including phylogenetic classification and screening for virulence factors. Using whole-genome sequencing, the isolate was identified as a *K. radicincitans* strain, revealing a virulence gene cluster for yersiniabactin biosynthesis in contrast to all other strains of the species. Whole-genome sequencing was the perfect method for identifying putative virulence factors of a particular *Kosakonia* strain and will help distinguish beneficial strains from pathogenic strains in the future. To our knowledge, this is the first report of *Kosakonia*-related bacteraemia from Europe.

## Introduction

*Kosakonia radicincitans* is a species within the new genus *Kosakonia*, until recently part of the genus *Enterobacter*. Many *Kosakonia* species have been isolated from plants and are known to improve plant performance^1,2^, but there are also rare reports that assume that some *Kosakonia* spp., such as *Kosakonia cowanii*, can act as facultative human pathogens^3^. There are also rare reports of *Kosakonia radicincitans* involved in human infections; however, all these reports are from outside Europe (mainly Asia and America). The first case of a human bloodstream infection with *K. radicincitans* was reported in a 61-year-old man with cholangiocarcinoma in Houston, Texas, USA, in December 2016^4^. However, the actual number of infections could have been underestimated, as this genus is relatively new and is therefore probably not yet included in all automated databases of bacterial diagnostic tools. It seems that *Kosakonia* has no higher pathogenic or resistance properties than *Enterobacter*, making a more precise diagnosis unnecessary in many cases^4^.

Therefore, this study used the rare opportunity to diagnose such an infection by careful evaluation. The aim of the study was to analyse the genetic background of a *Kosakonia* isolate from a patient with bacteraemia, including phylogenetic classification and screening for virulence factors.

Description of the case: An 85-year-old woman presented at the Department of Internal Medicine, in Graz, Austria, with icterus, occasionally diffuse abdominal pain and loss of appetite. Upon admission, she was afebrile, showed atrial flutter (3:1), and a heart rate of 100/min; she was in slightly poor general condition and had pressure pain in the middle and lower abdomen. Her liver values were significantly increased. She was diagnosed with bile duct stenosis and received a stent by endoscopic retrograde cholangiopancreatography (ERCP) on the third day of hospitalization. Four weeks after admission, the inflammation values increased, and she developed a fever. Two pairs of blood cultures were taken during the increase in inflammation parameters. One aerobic blood culture was positive for a bacterium initially identified as a member of the *Enterobacter cloacae* complex. Treatment with piperacillin/tazobactam (Pip/Taz) was initiated, and moxifloxacin was added later. Under treatment with this regime, the patient’s condition improved, and her inflammation parameters declined, and after six weeks of hospitalization, she was discharged.

## Results

### Microbiological testing

**Figure 1:**
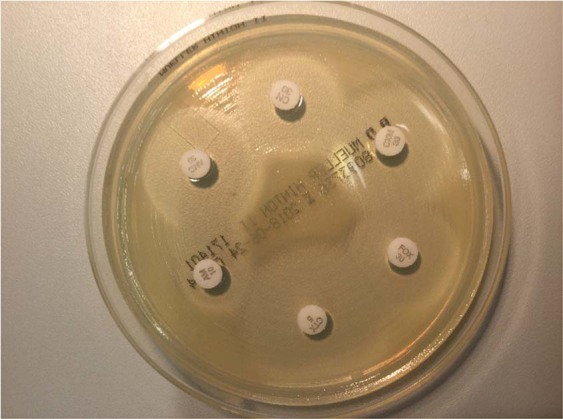
Resistance pattern of the *Kosakonia* isolate, which is atypical for *Enterobacter cloacae* ssp. *cloacae*. Ampicillin (AM), amoxicillin/clavulanic acid (AMC), cefalexin (CN), cefuroxime (CXM), cefoxitin (FOX) and cefotaxime (CTX).

Of the two sets of blood cultures obtained during the increase in inflammation parameters, one aerobic blood culture showed positive growth 20 hours after inoculation. Using Gram staining, the bacteria were shown to be gram-negative bacilli. Agar diffusion test and subcultures were performed and revealed the growth of grey-coloured colonies on blood agar and pink colonies on MacConkey agar. The agar diffusion test showed pan-susceptibility, with the exception of amoxicillin (Fig. 1, Suppl. Table 1). For identification, colonies from different agar media were subjected to MALDI-TOF (VITEK MS, bioMerieux), but the identification failed three times. Therefore, GN (gram-negative) and N196 automated biochemical testing was performed using the VITEK 2 system (bioMerieux), which yielded a 91% probability match with the *Enterobacter cloacae* complex. According to EUCAST (The European Committee on Antimicrobial Susceptibility Testing) guidelines, however, this genus was not found to be susceptible (intrinsic resistance to amoxicillin, amoxicillin/clavulanic acid and cefuroxime).

### Sanger sequencing and taxonomic classification

**Figure 2:**
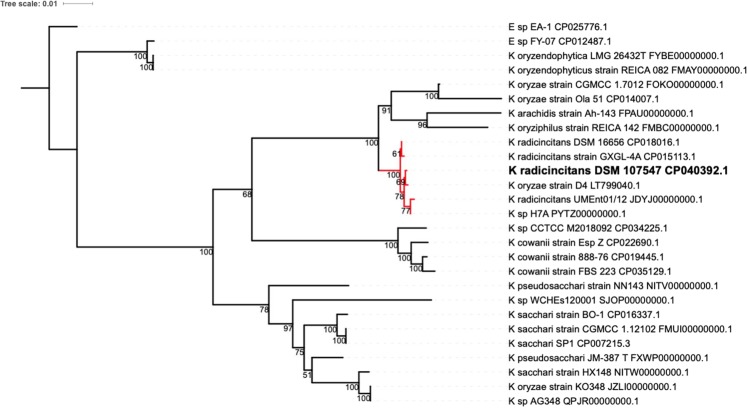
Best-scoring ML tree of 4 concatenated phylogenetic marker genes (*atp*D, *gyr*B, *inf*B, *rpo*B) confirms classification as *K. radicincitans* DSM107547 (bold). The *K. radicincitans* clade is highlighted in red. *Enterobacter* sp. EA-1 was used as an outgroup. Branch lengths indicate the number of substitutions per site. Bootstrap values are placed at internal nodes.

Accordingly, four housekeeping genes were analysed by Sanger sequencing, and the complete genome was sequenced for improved identification. All four housekeeping genes showed the highest similarity with sequences of *Kosakonia oryzae* strain D4 (ID: LT799040.1), namely, *atp*D (640/642 base pair identity), *gyr*B (688/688), *inf*B (611/612) and *rpo*B (635/637), and of *Kosakonia radicincitans* DSM16656 (ID: CP018016.1), namely, *atp*D (640/642), *gyr*B (688/688), *inf*B (609/612) and *rpo*B (637/637). According to these sequence analyses, the isolate is one of these two species. However, the strains cluster together with *K. oryzae* strain D4 (Fig. 2).

### Genome annotation

**Figure 3:**
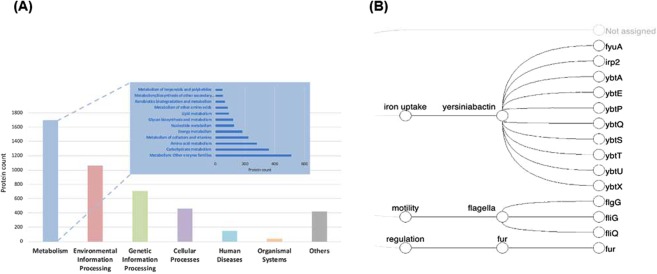
(**A**) Functional annotation of *K. radicincitans* DSM 107547 based on KEGG’s classification system (64% annotation rate) and (**B**) protein hits to virulence factors (MEGAN VFDB viewer, 94% identity threshold).

Mapping the proteins against KEGG’s KO identifiers (K numbers) provides assignments for the main functional categories: Metabolism (37.44%), Environmental Information Processing (23.44%), Genetic Information Processing (15.59%), Cellular Processes (10.1%), Human Diseases (3.26%), Organismal Systems (0.93%) and Others (9.24%; Fig. 3A). Potential virulence of the strain based on genomic features was estimated by comparison against the VFDB 2019^5^ (http://www.mgc.ac.cn/VFs/) and the web tool PathogenFinder 1.1 (https://cge.cbs.dtu.dk/services/PathogenFinder/; Table 1)^6^. Diamond protein alignment against the VFDB identified 14 potential virulence factors (identity threshold 94%), of which 10 proteins form the yersiniabactin iron uptake system, 3 are involved in flagellum biosynthesis and 1 is responsible for iron uptake regulation (Table 1), as visualized with the MEGAN6^7,8^ VFDB viewer in Fig. 3B. PathogenFinder predicted the strain as being “human pathogenic” with a probability of 71% due to the assignment of 54 genes to known pathogenic protein families with a median identity value of 96% (identity threshold 94%), among them 6 hits to yersiniabactin related genes. Additionally, 15 antimicrobial resistance genes could be detected with the CARD 3.0.2 - RGI 5.0.0 (Resistance Gene Identifier) web portal (Table 1, Suppl. Table 2).

**Table 1:** Genomic characteristics of *Kosakonia radicincitans* DSM 107547.

Features	*K. radicincitans* DSM 107547	Info (threshold: th.)
Genome	Chromosome (5,656,428 bp) Plasmid (118,312 bp)	CP040392 CP040393
Genes	5,487	NCBI PGAP
CDS	5,253	NCBI PGAP
RNA	116 (22 rRNAs, 83 tRNAs, 11 ncRNAs)	NCBI PGAP
KEGG	3,387	MEGAN: 70%/90% identity/coverage th.
PathogenFinder	54	94% identity th.
VFDB Hits	14	MEGAN: 94% identity th.
CARD Hits	15	Strict mode

## Discussion

Here, we report the first case of bacteraemia with *K. radicincitans*, previously known as *Enterobacter radicincitans*, in Europe. Some bacteria of the genus *Enterobacter* are major causes of human infections. However, the classification of *Enterobacter* species has changed rapidly in recent years. Several species were transferred to or excluded from this genus. In 2013, *Enterobacter* was divided into 5 new genera: *Lelliottia, Pluralibacter, Kosakonia, Cronobacter* and *Enterobacter*^9^. *Kosakonia* spp. (*K. radicincitans, K. sacchari, K. oryzae, K. cowanii, K. arachidis*) are usually known as plant growth-promoting bacteria, improving the yield and quality of fruits such as maize, radish, sugarcane or cabbage^1,2,10,11^. There are rare reports of *Kosakonia* spp. involved in human infections, but there are known species that can act as human pathogens, such as *Kosakonia cowanii*^3^. To date, there are no epidemiological data on the occurrence of *Kosakonia* species in human samples. The first case of a human bloodstream infection with *K. radicincitans* was reported in a 61-year-old man with cholangiocarcinoma in Houston, TX, USA, in Dec. 2016^4^. Unfortunately, the authors of this report considered some common features of enteric bacteria to be virulence factors, and confused type IV secretion systems with type IV pili that are involved in several phenomena, not only pathogenicity^12^.

Comparison of the cases from Austria and the USA showed that both patients had problems in the bile duct system. The patient in the USA presented with cholangiocarcinoma and fever, and the patient in Austria presented with bile duct stenosis and fever. Nevertheless, with only two cases considered, this could also be pure coincidence.

Future genomic comparisons will show whether human pathogenic strains can be clearly distinguished from plant-associated strains of the same bacterial species based on true virulence factors, such as the syntenic yersiniabactin-like gene cluster encoding an iron, copper and nickel ion-chelating siderophore^13^, which has been solely found in strain DSM 107547 among *Kosakonia* spp. Such a finding simplifies the diagnosis of pathogenic bacteria significantly. The role of yersiniabactin (Ybt) in mediating the virulence of human pathogenic bacteria is unquestionable: (i) invasive enteric bacteria from the genera *Yersinia*, *Escherichia*, and *Klebsiella* secrete Ybt during human infection to combat host-mediated metal deficiencies^13^; (ii) comparative genomics revealed that the list of horizontally transferred gene sets in *Salmonella enterica* is dominated by virulence factors and the yersiniabactin gene cluster^14^, suggesting an important role of this siderophore in human pathogenicity; and (iii) in addition to uropathogenic enterobacteria expressing Ybt^13^, it was shown very recently that all isolates of *Klebsiella pneumoniae* from infant blood or stool samples taken during outbreaks in neonatal intensive care units produced Ybt^15^.

Regarding the current *Kosakonia* infections, it must be considered that some infections with this organism were not correctly diagnosed previously. The most important concern is that diagnoses may be imprecise and may provide no or even false positive results: *Kosakonia* sp. yields no ID in MALDI-TOF analysis, and Vitek2 susceptibility testing suggests the presence of *Enterobacter* spp. A growing database for MALDI-TOF MS might solve this problem, as would the use of different MALDI-TOF systems. A second look at unsuitable germs and the resistance would also be useful with regard to the increased use of automation in many laboratories, where a specific pathogen is inevitably assigned to a stored antibiogram. We would like to motivate colleagues to take a closer look at such germs and publish the sequences of more of these isolates to facilitate further genomic comparisons of human samples and perhaps to test therapeutic options or antibiotic efficacy, as well as to obtain actual epidemiological data.

However, sequencing approaches for the identification of these bacterial species are recommended. Especially in our case, whole-genome sequencing is the perfect means for species identification and might help reveal new *Kosakonia* species in the future.

With improved diagnostic tools, researchers will be able to show whether infections with *Kosakonia* spp. are rare events indeed or occur more frequently than previously shown.

## Methods

### Strain

The *Kosakonia radicincitans* strain used in this study was archived at the DSMZ (German Collection of Microorganisms and Cell Cultures) as *Kosakonia radicincitans* strain DSM 107547.

### Microbiological methods

For identification, colonies from different agar media were subjected to MALDI-TOF (VITEK MS – bioMerieux), and N196 automated biochemical testing was performed using the VITEK 2 system (bioMerieux).

Susceptibility testing was performed as recommended by the European Committee on Antimicrobial Susceptibility testing (EUCAST)^16^. Interpretation of zone diameters was performed according to EUCAST 2017.

The following antibiotics were used: ampicillin (10 μg), amoxicillin/clavulanic acid (20 μg/10 μg), piperacillin/tazobactam (100 μg/10 μg), cefalexin (30 μg), cefuroxime (30 μg), cefoxitin (30 μg), cefotaxime (5 μg), ceftazidime (10 μg), cefepime (30 μg), imipenem (10 μg), meropenem (10 μg), amikacine (30 μg), gentamicin (10 μg), trimethoprim/sulfamethoxazole (1.25 μg/23.75 μg), ciprofloxacin (5 μg), and moxifloxacin (5 μg) (Becton Dickinson and Company, Sparks, MD, USA, BD BBL™). Sensi-DiscTM paper discs (BD) were used.

### Sanger sequencing and taxonomic classification

Identification based on sequencing of single marker genes was performed according to Brady *et al.*^9^, including the sequences of four housekeeping genes: *atp*D, *gyr*B, *inf*B and *rpo*B. For better classification, a phylogenetic maximum likelihood (ML) tree was inferred based on the MUSCLE v3.8.31^17^ alignment of the concatenated sequences of these housekeeping genes against closely related species (Fig. 2). The alignment was further trimmed by *trim*Al v1.2^18^ to remove all sites, obtaining more than 20% gaps, and more than 60% of the sites may be considered to be conserved. The final best-scoring tree was achieved with RAxML v8.2.12^19^, applying the GTRCAT approximation and rapid bootstrapping on 1000 replicates. The final visualization was performed with iTol v4.4.1^20^. Due to the very low distance of the strain to the subclade dominated by *K. radicincitans* (highlighted in red), supported with bootstrap values between 69% and 100%, the current affiliation to *K. radicincitans* is reasonable.

### Whole-genome sequencing

Whole-genome sequencing was performed using a combination of Pacific Biosciences long-read sequencing and Illumina short-read sequencing. For both sequencing runs, DNA was isolated using Qiagen Genomic-tip 100/G (Qiagen, Hilden Germany) according to the manufacturer’s instructions. The SMRTbell™ template library was prepared according to the instructions from Pacific Biosciences (Menlo Park, CA, USA), following the manufacturer’s Procedure & Checklist – Greater Than 10 kb Template Preparation. Briefly, for preparation of 15 kb libraries, 8 µg of genomic DNA was sheared using g-tubes™ from Covaris (Woburn, MA, USA) according to the manufacturer´s instructions. The DNA was end-repaired and ligated overnight to hairpin adapters by applying components from the DNA/Polymerase Binding Kit P6 from Pacific Biosciences (Menlo Park, CA, USA). Reactions were carried out according to the manufacturer´s instructions. BluePippin™ size-selection to greater than 4 kb was performed according to the manufacturer´s instructions (Sage Science, Beverly, MA, USA). Conditions for annealing of sequencing primers and binding of polymerase to the purified SMRTbell™ template were assessed with the Calculator in RS Remote (Pacific Biosciences, Menlo Park, CA, USA). One SMRT cell was sequenced on the PacBio RSII instrument (Pacific Biosciences, Menlo Park, CA, USA), taking one 240-minute movie. Libraries for sequencing on the Illumina platform were prepared by applying the Nextera XT DNA Library Preparation Kit (Illumina, San Diego, USA) with modifications according to Kishony *et al.*^21^. Samples were sequenced on a NextSeq™ 500 instrument. Genome assembly was performed by applying the RS_HGAP_Assembly.3 protocol included in SMRT Portal version 2.3.0 using default parameters. The assembly revealed a circular bacterial chromosome and a plasmid, both with coverages of 110× . Both replicons were circularized, artificial redundancies at the ends of the contigs were removed and adjustment to *dna*A (*par*A = soj) as the first gene was performed. Error correction was performed by mapping Illumina short reads onto the finished genome using Burrows-Wheeler Alignment (bwa 0.6.2) in paired-end (sample) mode using the default setting^22^ with subsequent variant and consensus calling using VarScan 2.3.6 (parameters: mpileup2cns–min-coverage 10–min-reads2 6–min-avg-qual 20–min-var-freq. 0.8–min-freq-for-hom 0.75–p-value 0.01–strand-filter 1–variants 1–output-vcf 1)^23^. A consensus concordance of QV60 could be confirmed.

The genome sequence has been deposited at NCBI GenBank under accession nos. CP040392 and CP040393 for the circular chromosome (5,656,428 bp) and plasmid, respectively (118,312 bp: Table 1).

Automated genome annotation, carried out with the NCBI Prokaryotic Genome Annotation Pipeline (PGAP), revealed a total of 5,487 genes, of which 5,253 are protein-coding genes, 116 RNAs and 118 pseudo-genes (Table 1). Mapping the proteins against KEGG’s KO identifiers (K numbers) using and known virulence factors of the VFDB 2019^5^ (http://www.mgc.ac.cn/VFs/) Diamond^24^ v.0.9.24 aligner and MEGAN v6.15.2 provided assignments for the main functional categories.

## Supplementary information

Supplementary information

Supplementary information2

Supplementary information3
