# Supplementary material for: First Report of Kosakonia radicincitans Bacteraemia from Europe (Austria) - Identification and Whole-Genome Sequencing of Strain DSM 107547
Source: Sci Rep. 2020 Feb 6;10:1948. doi: 10.1038/s41598-020-58689-x (PMC7005327; doi:10.1038/s41598-020-58689-x)
Supplement: Supplementary file 3 — Supplementary information3 [file 41598_2020_58689_MOESM3_ESM.docx]

**First Report of *Kosakonia* *radicincitans* Bacteraemia from Europe (Austria) - Identification and Whole-Genome Sequencing of Strain DSM 107547**

Tanja Mertschnigg^1^, Sascha Patz^2^, Matthias Becker^3^, Gebhard Feierl^1^, Silke Ruppel^4^, Boyke Bunk^5^, Cathrin Spröer^5^, Jörg Overmann^5, 6^, Gernot Zarfel^1*^

^1^Institute of Hygiene, Microbiology and Environmental Medicine, Medical University of Graz, Austria

^2^Algorithms in Bioinformatics, Center for Bioinformatics, University of Tübingen, Tübingen, Germany

^3^Institute for National and International Plant Health, Julius Kühn Institute, Federal Research Centre for Cultivated Plants, Braunschweig, Germany

^4^Leibniz Institute of Vegetable and Ornamental Crops, Grossbeeren, Germany

^5^Leibniz Institute DSMZ-German Collection of Microorganisms and Cell Cultures, Braunschweig, Germany

^6^Microbiology, Braunschweig University of Technology, Braunschweig, Germany

*Corresponding author: Institute of Hygiene, Microbiology and Environmental Medicine, Medical University of Graz, Neue Stiftingtalstrasse 6a, 8010 Graz, Austria.
Tel.: +43-316-385-73604, Fax: +43-316-385-79647, E-mail address: gernot.zarfel[@medunigraz.at](mailto:franz.reinthaler@medunigraz.at)
